# Metabolic Reprogramming in Cancer Cells: Emerging Molecular Mechanisms and Novel Therapeutic Approaches

**DOI:** 10.3390/pharmaceutics14061303

**Published:** 2022-06-19

**Authors:** Carla Navarro, Ángel Ortega, Raquel Santeliz, Bermary Garrido, Maricarmen Chacín, Néstor Galban, Ivana Vera, Juan Bautista De Sanctis, Valmore Bermúdez

**Affiliations:** 1 Endocrine and Metabolic Diseases Research Center, School of Medicine, University of Zulia, Maracaibo 4004, Venezuela; cpnm24@gmail.com (C.N.); angelort94@gmail.com (Á.O.); gabrielasanteliz29@gmail.com (R.S.); bermarygarrido@gmail.com (B.G.); nestorag17@gmail.com (N.G.); ivanaa19.iv@gmail.com (I.V.); 2 Universidad Simón Bolívar, Facultad de Ciencias de la Salud, Barranquilla 080002, Colombia; m.chacin@unisimonbolivar.edu.co; 3 Institute of Molecular and Translational Medicine, Faculty of Medicine and Dentistry, Czech Advanced Technology and Research Institute, Palacký University Olomouc, 779 00 Olomouc, Czech Republic; sanctisj@gmail.com

**Keywords:** metabolic reprogramming, tumor microenvironment, energy metabolism, neoplasms, carbohydrates, inflammation, immunotherapy

## Abstract

The constant changes in cancer cell bioenergetics are widely known as metabolic reprogramming. Reprogramming is a process mediated by multiple factors, including oncogenes, growth factors, hypoxia-induced factors, and the loss of suppressor gene function, which support malignant transformation and tumor development in addition to cell heterogeneity. Consequently, this hallmark promotes resistance to conventional anti-tumor therapies by adapting to the drastic changes in the nutrient microenvironment that these therapies entail. Therefore, it represents a revolutionary landscape during cancer progression that could be useful for developing new and improved therapeutic strategies targeting alterations in cancer cell metabolism, such as the deregulated mTOR and PI3K pathways. Understanding the complex interactions of the underlying mechanisms of metabolic reprogramming during cancer initiation and progression is an active study field. Recently, novel approaches are being used to effectively battle and eliminate malignant cells. These include biguanides, mTOR inhibitors, glutaminase inhibition, and ion channels as drug targets. This review aims to provide a general overview of metabolic reprogramming, summarise recent progress in this field, and emphasize its use as an effective therapeutic target against cancer.

## 1. Introduction

Cancer is a term used to define a group of diseases characterized by abnormal cell growth with an autonomous and uncontrolled expansion [1]. It represents a significant and escalating public health issue, responsible for one in six deaths worldwide. In this regard, in 2020, 19.3 million new cases and 10.0 million deaths from cancer were estimated [2], numbers that continue to increase, such that by the year 2040, cancer could be diagnosed in more than 29.4 million people per year [3].

It is currently known that cancer cells develop numerous properties that differentiate them from non-cancerous cells. These properties are known as hallmarks and include active proliferation, evasion of growth suppressors, resistance to cell death, angiogenesis, invasion and metastasis, immune evasion, tumour-promoting inflammation, genomic instability, mutation, non-mutational epigenetic reprogramming, polymorphic microbiomes, phenotypic plasticity, and metabolic reprogramming [3,4]. The latter refers to the ability of cancer cells to modify their metabolism, increasing the absorption and use of carbohydrates, lipids, and proteins to provide a pro-tumorigenic response in the face of the acquisition and maintenance of malignant properties [5]. 

Many factors mediate metabolic reprogramming: oncogenes, growth factors, hypoxia-induced factors, and the dysfunction of tumor suppressor genes. These alterations cause alterations in cell metabolism, especially glucose, whose absorption rate drastically increases in cancer [6]. 

Metabolic reprogramming allows cancer cells to adapt to drastic changes in the tumor environment. Tumors adapt to conventional antineoplastic therapies by generating chemoresistance, residual disease, and tumor relapse [7]. Although its role is not fully understood, it is considered a promising therapeutic target in the fight against cancer. Therefore, this review summarises the metabolic changes produced in cancer cells and their relationship to adaptability, survival, and proliferation, along with the current evidence regarding the use of metabolic reprogramming as an effective therapeutic target for cancer.

## 2. A Look into the Energy Metabolism of the Normal and Cancerous Cell

The most effective way to produce energy in normal cells is based on mitochondrial oxidative phosphorylation. Carbohydrates, especially glucose, are converted to pyruvate, the essential source of acetyl-coenzyme A (CoA). Likewise, proteins and lipids are catabolized to intermediates to maintain the tricarboxylic acid (TCA) cycle [8]. 

All cells need a source of energy to maintain homeostasis; however, cancer cells have additional energy requirements due to their constant growth and division. Tumor cells require large amounts of nutrients and energy to replicate. The necessities include deoxyribonucleic acid (DNA), ribonucleic acid (RNA), proteins, and lipids [9]. The glucose uptake rate tends to increase dramatically in parallel with the production of lactate instead of pyruvate, even in aerobic conditions and with a fully functioning mitochondrial metabolism; this phenomenon is known as the Warburg effect [10]. 

The Warburg effect shows the ineffective production of energy. Under aerobic conditions, 36 adenosine triphosphate (ATP) molecules are produced per glucose molecule. A cancer cell is in constant proliferation; thus, it needs to maintain high levels of glycolytic intermediates, such as fructose-6-phosphate and glyceraldehyde-3-phosphate, to generate ribose-5-phosphate destined for the synthesis of nucleotides and NADPH. NADPH is used as a cofactor by glutathione reductase (GR) to reduce glutathione (GSH), and thus, reduce reactive oxygen species (ROS); it is also required for de novo synthesis of non-essential amino acids and the synthesis of acetate for the production of fatty acids [11]. 

On the other hand, the accumulation of oncometabolites—lactate, glutamate, fumarate, and succinate—constitute a critical influence on cancer cell metabolism, increasing cell survival and progression [12]. The high expression of glycolytic enzymes, glutaminolysis, and the presence of a large number of metabolic transporters is associated with elevated lactate production, which stimulates angiogenesis by various mechanisms. Lactate activates the NF-κB/IL-8 pathway in endothelial cells for the formation of new blood vessels; at the same time, the expression of the lactate dehydrogenase (LDH) isoform is related to the production, by endothelial cells, of hypoxia-inducible factor (HIF)-1α/HIF-2α and vascular endothelial growth factor (VEGF), essential mediators of the angiogenesis process. Abnormal angiogenesis is also stimulated by tumor-associated macrophages (TAMs), activation of (mTOR) complex 1 (C1), ERK1, STAT3, via HIF-1α ubiquitination, and the up-regulation of carbonic-anhydrase (CA) 9 gene expression, and DNA damage responses 1 (Redd1).

There are instances during cancer where lactate is not only produced by cancer cells but also by cancer-associated fibroblasts (CAFs) through ROS-stimulated glycolysis [13]. The excess lactate produced can enter the cancer cell through monocarboxylate transporter 1 (MCT-1). This event is known as the reverse Warburg effect, and it allows the cells to be independent of the need for glucose as the sole source of energy [14].

In contrast, the oncometabolite glutamate, a result of glutamine metabolism, is converted to α-ketoglutarate and incorporated into the TCA cycle. Glutamine is a product of glutamine synthetase (GS) activity and can be transformed into lactate in the cytosol [15]. In cancer cells, an increase in lactate concentration and HIF1 activation causes a shift in the direction of metabolic reactions in the Krebs cycle to depend on glutamate by inhibiting pyruvate dehydrogenase (PDH) and pyruvate carboxylase (PC). Tumors use glutamine as their energy source, resulting in the accumulation of citrate and malate to increase the synthesis of nucleotides destined for the growth of neoplastic cells. 

Furthermore, the IDH1-mediated reductive carboxylation pathway is able to generate 2-hydroxyglutarate from glutamine via alpha-ketoglutarate; in turn, citrate, also derived from glutamine, supports fatty acid synthesis. In addition, this phenomenom also helps to eliminate ROS generated in tumor cells through the generation of NADH from IDH1-dependent glutamine reductive metabolism [16].

In a similar manner, lipids play a major role during cancinogenesis by increasing fatty acid uptake through the expression of specialized transporters. Together with augmented de novo lipogenesis, beta oxidation of fatty acids, and altered lipid storage, lipids are related to a more aggressive phenotype, resulting in increased cell migration and evasion of apoptosis [17,18].

## 3. Metabolic Pathways in Cancer Cells

Metabolic reprogramming is a common and essential cancer feature. Contrary to what has been considered in the past, this process is not stable and tends to vary according to the availability of substrates and the different metabolic demands for cell proliferation, growth, invasion, and survival [19]. Cancer cells alter their ability to metabolize carbohydrates, lipids, and proteins to adapt to cellular demands, environmental conditions, and changing accessible substrates [20]. However, substrate unavailability is not the only inducer of this hallmark. It has been shown that the tumor microenvironment plays a significant role in the metabolic reprogramming of cancer cells and tumor environment-dependent adaptation [21].

### 3.1. Carbohydrate Metabolism

**Figure 1:**
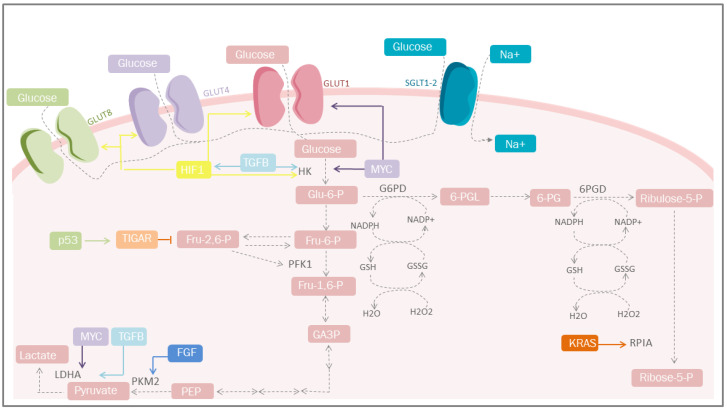
Carbohydrate metabolism and mediators of metabolic reprogramming. Cancer cells must acquire a greater amount of nutrients, especially glucose. One of the mediators is HIF-1, which increases glucose uptake through the induction of GLUT-1, GLUT-4 and GLUT-1; simultaneosly, it can also be stimulated by TGF-β through the PI3K/AKT/mTOR pathway. Other important mediators are p53 that plays a protective role against ROS. GLUT, glucose transporters; Glu-6-P, glucose 6-phosphate; Fru-6-P, fructose 6-bisphosphate; Fru-1,6-P, fructose 1,6-bisphosphate; Fru-2,6-P, fructose 2,6-bisphosphate; GA-3-P, glyceraldehyde 3-phosphate; PEP, phosphoenolpyruvate; HK, hexokinase; TGFB, transforming growth factor beta; PFK1, phosphofructokinase 1; PKM2, pyruvate kinase M2; LDHA, lactate dehydrogenase A; G6PD, glucose-6-phosphate dehydrogenase; 6-PGL, 6-phosphogluconolactonase; NADP+, nicotinamide adenine dinucleotide phosphate; NADPH, reduced form of NADP; GSSG, glutathione disulfide; GSH, glutathione; H_2_O_2_, hydrogen peroxide; TIGAR, Tp53-induced glycolysis and apoptosis regulator; FGF, fibroblast growth factor; HIF-1, hypoxia-inducible factor 1; PPP, pentose phosphate pathway; KRAS, Kirsten-ras; RPIA, ribose-5 phosphate isomerase; MYC, proto-oncogene; Ribulose-5-P, ribulose 5-phosphate; Ribose-5-P, ribose 5-phosphate; SGLT1/2, sodium-glucose cotransporter-1/2.

Increased glucose uptake and lactate production in the presence of oxygen, regardless of the anabolic and catabolic needs of cancer cells, has been recognized as a cancer trademark for nearly a century. This is reflected in the coupling of NAD/NADH between glyceraldehyde phosphate dehydrogenase and LDH [22]. On the other hand, mitochondrial uncoupling in cancer cells represents an important source of NAD in the cytosol, increasing glycolytic efflux. An incremented flow of glucose provides intermediaries for different cellular pathways, among which the PPP pathway stands out, since it is the most studied, where G6PD and 6-phosphogluconate dehydrogenase participates in redox homeostasis through the generation of GSH [23] and the synthesis of glycerol-3-phosphate for lipid biosynthesis (Figure 1) [24]. However, other critical metabolic pathways exist, including nuclear glycogen metabolism, which is increased in specific cancer subtypes [25], and gluconeogenesis, which plays an essential role during glucose deprivation through the metabolic flexibility conferred by PCK1 or PCK2, promoting cancer cell survival [26].

In the absence of glucose, cancer cells overexpress phosphoenolpyruvate carboxykinase, initiate gluconeogenesis, and metabolize glucose [27]. Other carbohydrates that play essential roles during cancer stages include hexosamine and fructose. Hexosamine promotes EMT by increasing the nuclear location of beta-catenin by activating its pathway through the glutamine-fructose-6-phosphate transaminase [28]. In contrast, fructose drives metabolic reprogramming by promoting central carbon metabolism via aldolase B to induce metastasis [29].

### 3.2. Lipid Metabolism

Even though the most studied aspect of metabolic reprogramming concerns glucose, the altered metabolism of lipids has received much attention due to their essential role as structural components of the cellular membrane, second messengers, and a source of fuel for energy production [30].

**Figure 2:**
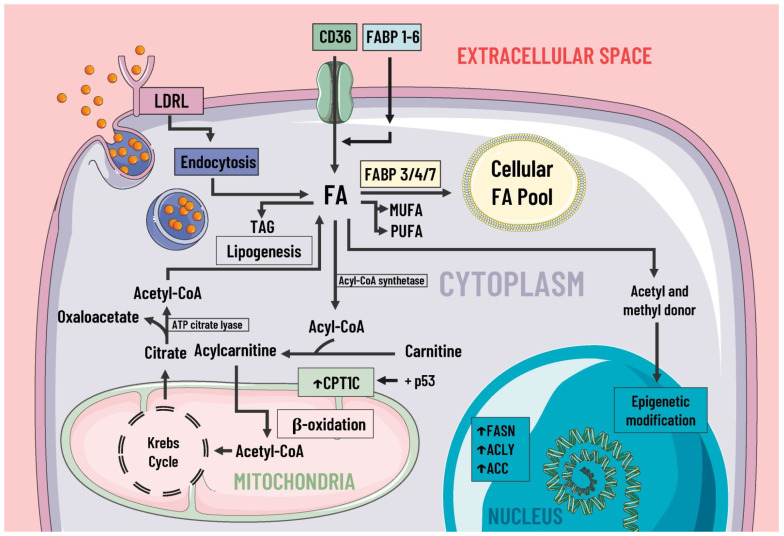
Lipids metabolism and mediators of metabolic reprogramming. Lipid metabolism in cancer cells is altered to increase the availability of these molecules, which provide structural components for the cell membrane, second messengers, and a fuel source. Fatty acids enter the cell by several pathways, including LDRL-mediated endocytosis and a wide variety of membrane transporters, such as CD36 and FABP1-6. Another source of FAS is lipogenesis. Once in the intracellular space, FAS can be stored in the cellular FAS pool by means of FABP 3/4/7, and can also serve as a substrate for the formation of MUFA, PUFA, TAG, etc. Likewise, some authors suggest FAS are a great energy source through β-oxidation and act as donors of acetyl and methyl groups, participating in epigenetic modifications. In cancer, there is an increase in the expression of enzymes involved in lipogenesis, cholesterol synthesis, and beta-oxidation, such as ACC, FASN, ACLY, and CPT1C, where the latter is due to p53. LDRL, low-density lipoprotein receptor; CD36, a cluster of differentiation 36; FABP, fatty acid-binding protein; FAS, fatty acids synthetase; MUFA, monounsaturated fatty acids; PUFA, polyunsaturated fatty acids; TAG, triacylglycerides; CPT1C, carnitine palmitoyltransferase 1C; FASN, fatty acid synthase; ACLY, ATP citrate lyase; ACC, acetyl-CoA carboxylase; ↑, increase.

During cancer, reprogramming of lipid metabolism results from several processes, such as increased fatty acid uptake, de novo lipogenesis, beta-oxidation of fatty acids, and altered lipid storage [17]. In cancer lipid metabolism, a common feature is high levels of fatty acid uptake due to the presence of specialized transporters in the plasma membrane. CD36, fatty acid transport protein family (FATPs), and plasma membrane fatty acid-binding proteins (FABPpm) all exacerbate the aggressiveness, growth, and survival of cancer cells [17] (Figure 2).

CD36 facilitates the uptake of fatty acids by tumor cells, enhances lipid droplet (LD) development, and promotes the formation of acylcarnitine, monoacylglycerols, and other phospholipids [31]. It also facilitates fatty acid oxidation (FAO) [31]. The increase in fatty acid uptake hampers antigen presentation. Considering its high heterogeneity and binding capacity for different ligands, CD36 is intimately related to the presence of thrombospondin-1 (TSP-1), known for its antitumorigenic effects. Current evidence has shown that it also plays an essential part in cancer development by promoting the formation of an embolus of tumor cells and cell adhesion. In addition, it induces the release of TGF-β, promoting tumor growth by altering angiogenesis through the modulation of proangiogenic proteins, such as JNK and p38a [31,32].

In cancer cells, lipogenesis parallels elevated FAS expression, cell migration, evasion of apoptosis, and aggressiveness [33,34]. The accumulation of fatty acids by lipogenesis results in their storage in LDs, and cytoplasmic lipases catalyze their output. Adipose triglyceride lipase (ATGL), hormone-sensitive lipase (HSL), and monoacylglycerol lipase (MAGL) sustain tumor survival [18].

The activation of lipases and the availability of fatty acids increases the rate of electron flow. It produces an increase in the formation of ROS, which have multiple protumorigenic effects by facilitating cell proliferation, tumor growth, and adaptation through their participation in multiple signaling pathways, among which stand out the following: the mitogen-activated protein kinase (MAPK) cascades, phosphoinositide-3-kinase (PI3K)/Akt, and the nuclear factor kappa light chain-enhancer of activated B cell (NF-kB) pathways. Therefore, the correlation between lipid catabolism and ROS production is important in regulating cancer growth and progression [18,35].

All the effects mentioned above are augmented by the loss of p53 activity and mutations [36]. The upregulation of enzymes related to mevalonate anabolism are involved in cholesterol synthesis and protein prenylation [37].

### 3.3. Protein Metabolism

**Figure 3:**
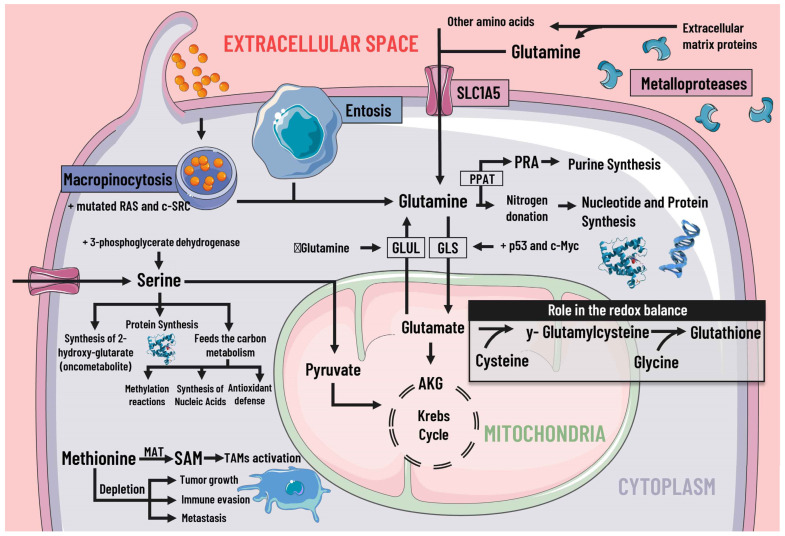
Protein metabolism and mediators of metabolic reprogramming. Protein metabolism in cancer cells is altered due to the immense demand generated by constant cell division, which is why amino acids are of great importance as proteogenic building blocks. Among the amino acids, glutamine is of great relevance in cancer cells. Glutamine enters the cell by different pathways, including macropinocytosis (which is increased by mutated RAS and c-SRC), entosis, degradation of cell-matrix proteins by metalloproteases, and transport by SLC1A5. Glutamine serves as a nitrogen donor and is used to synthesise nucleotides, purines, and proteins. It can also be a source of energy through glutaminolysis, which p53 and c-Myc stimulate. When there is a decrease in glutamine levels, it is synthesized by GLUL using glutamate as a substrate. Likewise, glutamine intervenes indirectly in the redox balance through glutamate. Serine is also essential, since increases in serine and 3-phosphoglycerate dehydrogenase, the first enzyme involved in its synthesis, are associated with tumor growth. Serine feeds carbon metabolism and protein synthesis, favors the synthesis of the oncometabolite 2-hydroxy-glutarate, and can act as an energy source, since it is an anaplerotic metabolite. In addition, methionine and SAM facilitate the pattern of histone methylation in monocytes/macrophages and the activation of TAMs. Depletion of exogenous methionine promotes tumor growth, metastasis, and immune evasion. SLC1A5 solute transporter family member 5; PPAT, phosphoribosyl pyrophosphate amidotransferase; PRA, phosphoribosylamine; GLS, glutaminase; GLUL, glutamine synthetase; AKG, alpha-ketoglutarate; SAMs S-adenosylmethionine; MAT, methionine adenosyltransferase; TAMs, tumor-associated macrophages.

Cancer cells adapt their metabolism to support biomass production; therefore, they have a high rate of protein synthesis. To maintain correct protein synthesis, the contribution of amino acids is vital as proteogenic building blocks. Among the most notable is glutamine, the most abundant non-essential amino acid in plasma and the main nitrogen donor for the synthesis of purines, pyrimidines, and NAD [38], so its deficiency leads to the interruption of protein synthesis [39] (Figure 3).

The high rate of glutamine consumption and uptake through the SLC1A5 transporter shows that cancer cells are dependent on this amino acid. Glutamine provides intermediates for the TCA cycle. In addition, it is also involved in protein synthesis by being the precursor of other amino acids, such as glutamate, asparagine, aspartate, and proline [40]. Some mediators can regulate glutamine metabolism, such as p53 and c-Myc, by stimulating GLS [41]. The deamination of glutamine leads to the release of ammonia. Ammonia is recycled for generating other amino acids, including aspartate, leucine, and proline, through GLUD [42]. When extracellular glutamine is scarce, cancer cells can increase their production through the enzyme glutamate-ammonia ligase (GLUL) [38].

Another amino acid of great importance in cancer development is arginine, an essential amino acid that participates in many metabolic pathways. In cancer cells, the enzyme argininosuccinate synthetase (ASS) (theh limiting enzyme for de novo arginine synthesis) is deficient, which leads the tumor cell to obtain exogenous arginine [43]. On the other hand, serine contributes to the donation of metabolites destined for the TCA cycle and favors the synthesis of oncometabolites [44]. Likewise, other essential amino acids, including aspartate and proline, support nucleotide biosynthesis and ATP generation [39].

Cancer cells use alternative processes for extracellular matrix (ECM) survival and proliferation [20]. These processes include the degradation of ECM proteins into peptides by matrix metalloproteases. Macropinocytosis is also used to internalize and degrade exogenous proteins and entosis. One living cell is completely internalized by another, where it is destroyed and digested by the host cell [45].

### 3.4. Methionine, S-Adenosylmethionine and Cysteine

Similarly, methionine and S-adenosylmethionine (SAM), a biological methyl donor generated from methionine by methionine adenosyltransferase (MAT) enzymes, facilitate histone methylation patterns in monocytes/macrophages as well as activation of tumor-associated macrophages (TAMs). It is known that depletion of exogeneous methionine or inhibition of MAT2A impairs M1 and M2 polarization of macrophages in addition to promoting tumor growth, metastasis, and immune evasion [46].

At the same time, the relationship between cysteine and cancer has been actively studied. It is acknowledged that sulfur and carbon metabolism reprogramming underlies the adaption to a hypoxic microenvironment promoted by cysteine. In the extracellular environment, cysteine is present in an oxidized dipeptide named cystine. Cystine uptake is driven by the cystine/glutamate antiporter xCT and immediately reduced to cysteine by NADPH. Furthermore, cysteine is synthesized de novo by the transsulfuration (TSS) pathway in cancer cells under cystine deprivation [47].

### 3.5. Isoenzymes

#### 3.5.1. Fumarate Hydratase (FH)

The enzyme FH is part of the TCA cycle, where it catalyzes the reversible formation of fumarate to malate. Its mutation or silencing contributes to carcinogenesis by compensating for mitochondrial deterioration by metabolic changes [48]. FH increases glycolytic flux. Instead of oxidizing glucose in the mitochondria, it is diverted to the PPP pathway, and the rest is converted into lactate through increased glycolytic enzymes and inhibition of PDH [49]. In addition, due to the decreased entry of glucose into the TCA cycle, it is replaced by glutamine as the primary carbon source to supply alpha-ketoglutarate and subsequently generate NADPH destined to participate in oxidative phosphorylation, along with the transformation to citrate for lipid biosynthesis thanks to reductive carboxylation [50].

The accumulation of intracellular fumarate can create a state of pseudohypoxia through HIF stabilization via the NF-kB pathway. The intracellular increase of this oncometabolite pressures cancer cells to metabolize arginine to form argininosuccinate by the enzyme argininosuccinate lyase (ASL) [50].

#### 3.5.2. Isocitrate Dehydrogenase

The enzyme isocitrate dehydrogenase (IDH) is responsible for catalyzing the oxidative decarboxylation of isocitrate to alpha-ketoglutarate and carbon dioxide (CO2) in the TCA pathway. The enzyme is involved in a series of metabolic functions, which includes glucose sensing, glutamine metabolism, lipogenesis, and regulation of the redox state [51].

An IDH mutation leads to major metabolic changes involving increased oxidative stress through the reduction of NADPH [52], lactate dehydrogenase A (LDHA) silencing (suggesting that the carbon source is preferentially derived from alpha-ketoglutarate via glutamine metabolism), and PDH suppression (via elevated PDK3 expression) accompanied by increased activity of PC, an essential enzyme for producing intermediate metabolites in various pathways crucial for gluconeogenesis and lipogenesis [53]. Likewise, an IDH mutation increases 2-hydroxyglutarate, a key metabolite during metabolic reprogramming [54].

#### 3.5.3. Glutaminase

Glutamine is one of the main energy sources used by cancer cells, right after glucose. Glutamine plays a part in metabolic pathways essential for growth, proliferation, and defence against oxidative stress [15]. Its metabolic products, glutamate and alpha-ketoglutarate, participate in synthesising fatty acids, transamination, amino acid synthesis, and TCA support. All the processes mentioned above are metabolized by GLS [55].

There are two GLS isoenzymes: GLS and GLS2. GLS, regulated by the MYC and mTORC-1 oncogenes, engages in macromolecule synthesis as well as energy supply through TCA glutaminolysis, whereas GLS2, regulated by p53, fulfils different roles in energy metabolism and antioxidant defence [56]. Since GLS catabolizes glutamine for the final generation of ATP and GSH, its reduction or silencing play a key role cell proliferation and survival, leading to cell death due to decreased ATP and excessive generation of ROS [57].

#### 3.5.4. Lactate Dehydrogenases

In cancer, most of the pyruvate generated by excessive glycolysis is diverted to the generation of lactate, even if oxygen is present, with the help of LDH. One way in which LDH is able to facilitate tumor progression is through interaction with the tumor stroma, where it is important for ECM remodeling, activation of signaling pathways, the release of inflammatory molecules, and fueling of CAFs, all thanks to the lactate generated in the tumor cell that subsequently released by increased expression of the lactate transporter MCT4. An increase in extracellular lactate also triggers an increase in the expression of MCT1 and LDH in the CAFs, which are then absorbed and converted into pyruvate to satisfy the needs of these stromal cells, thereby initiating positive feedback between cancer cells and the stroma [58].

## 4. Mediators of Metabolic Reprogramming

There are a large number of key regulatory factors that, as direct or indirect targets of mutations, can give way to the metabolic reprogramming of a cell and make it cancerous, since it allows them to cope with the stress produced by hypoxia and lack of nutrients in the tumor microenvironment. The latter is considered a heterogeneous and complex collection characterized by an acidic pH, hypoxia, increased interstitial pressure, and fibrosis. In addition, it is formed by acellular components such as the ECM and signaling molecules, together with cellular components such as fibroblasts, immune cells, endothelial cells, and adipocytes. It is important to understand that the tumor microenvironment is a continuously evolving entity, which varies with the type and stage of the tumor. During early tumor development, there is a dynamic interaction with the microenvironment. The microenvironment coordinates a program of mediators for its evolution and survival. These mediators include different growth factors, oncogenes, tumor suppressor genes, and enzymes, as discussed in the following sections [59,60].

### 4.1. Growth Factors

Among growth factors, the growth and differentiation factor transforming growth factor-beta (TGF-β) has a prominent role in carcinogenesis. Under physiological conditions, TGF-β is involved in regulating critical cellular functions, such as differentiation, proliferation, migration, apoptosis, immune surveillance, and maintenance of homeostasis [38]. Its dysregulation contributes to developmental abnormalities, fibrotic disorders, immune diseases, and tumorigenesis [39].

Regarding its role in cancer, it is known that TGF-β promotes angiogenesis and immune evasion, inducing a process called epithelial to mesenchymal transition (EMT) through activiation of the traditional mothers against decapentaplegic homolog (SMADs) pathway, which is important for stimulating invasiveness and cell survival [40]. At the same time, TGF-β is involved in metabolic reprogramming required to meet the increased energy needs of the EMT through alternative pathways, such as PI3K/protein kinase B (AKT) and p38 mitogen-activated protein kinases (p38 MAPK), which are responsible for the increased expression of the glucose transporter (GLUT) 1 [41].

In this sense, it has been observed that there is an activation of mTOR induced by TGF-β and the PI3K-Akt pathway in cancer cells, which regulates endothelial cell growth and catalyzes the production of 3′-phosphorylated phosphoinositides enhancing cancer stem cells characteristics [61]. In addition, the mTOR and PI3K pathways can lead to resistance to cancer cell apoptosis and induce EMT [62]. Together, mTOR expression has been associated with poor prognosis in some types of cancer, so mTOR inhibitors could represent a therapeutic alternative in order to improve the effectiveness of current treatments [63].

In turn, TGF-β induces necessary glycolytic enzymes, such as 6-phosphofructo-2kinase/fructose-2,6-biphosphatase 3 (PFKFB3), HK2, and LDH, resulting in a greater glycolytic flux [42]. At the same time, pyruvate dehydrogenase kinase 4 (PDK4), a negative regulator of PDH, is positively regulated by TGF-β, which further favors the glycolytic phenotype, lactate production, and confers resistance to apoptosis.

Similarly, TGF-β is involved in the regulation of lipid metabolism through protein kinase C (PKC)-dependent translocation of the fatty acid translocase CD36 (CD26/FAT) to the plasma membrane. CD36 favors the absorption of long-chain fatty acids, such as palmitate, which takes place in the mitochondria for oxidation and subsequent energy production. These fatty acids can also be stored as triacylglycerols in lipid droplets, serving as energy reserves for cancer cells [43]. Similarly, induction of the expression of stearoyl-CoA desaturase-1 (SCD1), a limiting enzyme for the transformation of saturated fatty acids into monounsaturated fatty acids, by TGF-β has been observed in many types of cancer [44].

Like TGF-β, other less studied growth factors take part in cancer progression. These include epidermal growth factor (EGF), which promotes EMT through metabolic reprogramming, particularly increasing glycolytic metabolism via the epidermal growth factor receptor (EGFR)/PI3K/HIF-1 axis promoting PDK1 expression [45]. On the other hand, EGF favours glutamine to supply the acetyl-CoA necessary for lipogenesis, and modulates the synthesis of monounsaturated fatty acids through phosphorylation of SCD1 [48,64].

### 4.2. Hypoxia-Inducible Factors

**Figure 4:**
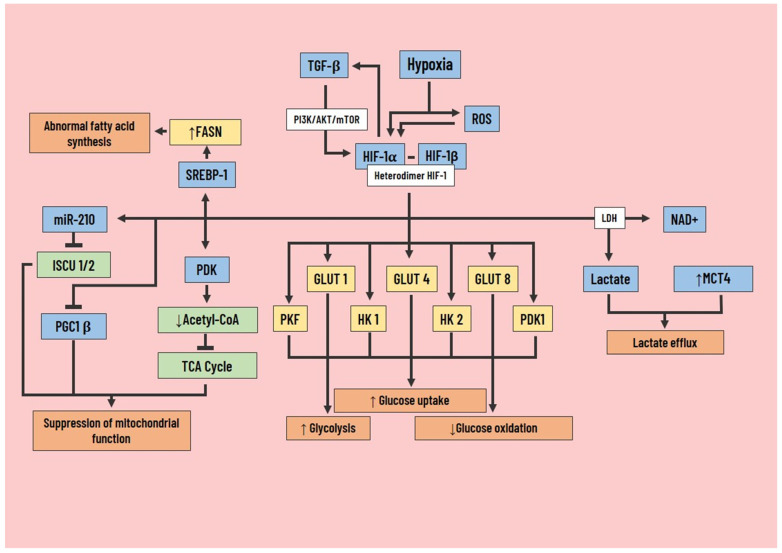
Role of hypoxia inducible factor-1 in cancer cell metabolic reprogramming. Hypoxia modifies cell metabolism through the expression of HIF-1α, which influences tumor angiogenesis, cancer cell migration, invasion, and glycolytic metabolism. In addition to this, HIF-1α can be activated by TGF-β action via the PI3K/AKT/mTOR pathway, and at the same time, HIF-1α can activate TGF-β in a positive feedback loop. Among the most important metabolic changes produced by HIF-1α are increased glucose uptake, glycolysis, and decreased glucose oxidation through the induction of GLUT 1/4/8, PKF, HK 1/2, and PGK1. There is also an increase in lactate and NAD+ production through LDH. Together, an increase in MCT4 expression contributes to lactate efflux. Another effect of HIF-1α expression is the suppression of mitochondrial function, resulting from inactivation of the TCA cycle, inhibition of PGC1 β, and repression of ISCU 1/2 by miR-210. Finally, HIF-1α promotes abnormal fatty acid synthesis through overexpression of FASN, which is mediated by increased SREBP-1 activation. HIF-1α, hypoxia-inducible factor-1; TGF-β, transforming growth factor beta; PI3K, phosphoinositol 3-kinase; LDH, lactate dehydrogenase; NAD, nicotinamide adenine dinucleotide; MCT4, monocarboxylate transporter 4; GLUT, glucose transporter; PKF, phosphofructokinase; PGK1, phosphoglycerate kinase 1; HK, hexokinase; PDK, phosphoinositide-dependent kinase; TCA, tricarboxylic acid; PGC1 β, peroxisome proliferator-activated receptor-gamma coactivator-1 beta; miR-210, microRNA-210; ISCU, iron-sulfur group; SREBP-1, sterol regulatory element binding protein 1; FASN, fatty acid synthase; ↑, increase; ↓, decrease.

Hypoxia is a microenvironmental factor that broadly influences the behavior of cancer and stromal cells to support metastasis, and it results in the expression of HIFs, a family of transcription factors that activate the genes that control angiogenesis, migration, invasion, and glycolytic metabolism. However, the HIF-1α can also be stimulated by TGF-β through the PI3K/AKT/mTOR pathway and mediate TGF-β-induced effects, becoming a feedback cycle [65] (Figure 4).

Although various studies recognize HIF-1α and HIF-2α as promoters at the onset of various tumor entities, their role during tumorigenesis is positively associated with malignant progression. In carcinomas that have lost and/or silenced the Von Hippel–Lindau (VHL) tumor suppressor gene, which is responsible for the destruction of HIF in the presence of oxygen, there is an increase in HIF. However, conflicting results make it difficult to understand their role in cancer [66].

In line with the pro-tumorigenic findings, HIF-1, regulated by the PI3K/AKT pathway, stimulates glucose uptake and glycolysis and significantly decreases glucose oxidation. This effect is achieved through the induction of GLUT1, GLUT4, and GLUT8, along with glycolytic enzymes hexokinase 1 and 2 (HK), pyruvate kinase (PK), phosphofructokinase (PKF), phosphoglycerate kinase 1 (PGK1) [67], and lactate formation by LDH [68]. The aforementioned, along with the increase in the expression of MCT4, participates in the efflux of lactate.

On the other hand, HIF-1 is responsible for the suppression of mitochondrial function through several pathways, the first being through the expression of PDK leading to a decrease in acetyl-CoA levels and inactivation of the TCA cycle [69]. The second is by induction of microRNA-210 (miR-210), which represses the assembly protein of the iron-sulfur group 1/2 (ISCU 1/2). The group is involved in the activation of important enzymes in mitochondrial activity [70]. The third is by indirect inhibition of PGC1β, a positive transcriptional regulator of mitochondrial biogenesis that is MYC dependent. HIF-1 is responsible for its modulation by inhibiting MYC activity [71].

In some hypoxic cancer cells, there is abnormal synthesis of fatty acids independent of extracellular availability thanks to the overexpression of FASN, a product of increased expression and activation of the sterol-regulating element-binding protein 1 (SREBP-1) due to HIF-1 [72]. In contrast, other studies suggest that there is greater absorption of exogenous fatty acids due to the decreased generation of glucose-based acetyl-CoA, elevated levels of genes involved in its storage (Perilipin 2 (PLIN2), and hypoxia-inducible lipid droplet associated protein (HILPDA). This indicates that HIF-1 improves lipid accumulation [73]. Alternatively, to compensate for the low availability of glucose-derived acetyl-CoA, cancer cells switch to other carbon sources, such as glutamine or acetate, for fatty acid synthesis [74].

### 4.3. Tumor Suppressor Genes

#### 4.3.1. P53

The transcription factor and tumor suppressor p53 is the most frequently mutated human gene in cance., It controls the expression of proteins involved in cell cycle arrest, DNA repair, apoptosis, and senescence. Simultaneously, it plays an important role in glycolytic and lipid metabolism, and the improvement of oxidative phosphorylation. However, mutation or deletion results in the loss of its functions, thus promoting metabolic reprogramming [75].

Under physiological conditions, p53 is known to repress GLUT1 and GLUT4 transcription to decrease glycolytic flux directly, and it represses GLUT3 expression through downregulation of activated NF-kB [76]. Moreover, it decreases the levels of key glycolytic enzymes, such as HK2, phosphoglycerate mutase 1 (PGM1), and PDK2 [77]. In addition, p53 is capable of repressing glycolysis through a protein called parkin that also functions as an activating factor for the degradation of HIF-1α [78], and to suppress MCT4 transcription [79].

Likewise, p53 plays a protective role against ROS-associated apoptosis by expressing a gene called regulator of apoptosis and glycolysis induced by TP53 (TIGAR). TP53 reduces the cellular levels of fructose-2,6-bisphosphate, resulting in a decrease in glycolysis and ROS by diverting glycolytic intermediates to the PPP [80]. In contrast, some studies have described inhibition of the PPP pathway thanks to a direct union with G6PD [81]. However, it should be noted that p53 and its relationship with cell cycle control are neither unique nor unidirectional.

In cancer cells, mutated p53 promotes lipid metabolism in various tumors by binding to and activating SREBP, inducing the expression of genes involved in lipid metabolism. In normal cells, p53 induces malonyl-CoA decarboxylase (MCD), which catalyzes the conversion of malonyl-CoA to acetyl-CoA, lipin1 and SIRT, preventing lipid accumulation [37].

In summary, p53 mutations lead to a reduction in all its activities, increasing glycolytic flux, the PPP pathway, and lipid metabolism in tumor cells [82], coupled with decreased mitochondrial respiratory activity through the loss of cytochrome C oxidase-2 (SCO2), an essential protein for the assembly and function of cytochrome C oxidase (COX) in the mitochondrial respiratory chain [80].

#### 4.3.2. Phosphatase and Tensin Homolog

The phosphatase and tensin homolog, or phosphatidylinositol-3,4,5-triphosphate 3-phosphatase (PTEN), is known as the most important negative regulator of the PI3K/AKT signaling pathway through PIP3 dephosphorylation. It is also associated with the jun N-terminal kinase (JNK) pathway, in parallel and independently of PI3K/AKT, so its loss is an important event in carcinogenesis [83]. Metabolic reprogramming produced by the mutation or deletion of PTEN induces malignant growth together with the proliferation of cancer cells by altering intracellular metabolism [84].

PTEN mutations have been shown to alter Akt activation that promotes GLUT1 expression, leading to elevated glucose uptake, lactate production [85], and HK2 upregulation [86]. Concurrently, there is an increase in fructose-2,6-bisphosphate, the most potent allosteric activator of PFK-1. Altogether, these changes contribute to the Warburg effect. In addition, thanks to the stabilization of GLS, glutaminolysis is significantly increased along with various metabolites, such as 2-ketoglutarate, malic acid, and fumaric acid [84].

Many types of cancers that have lost PTEN depend more on lipid metabolism than on anaerobic glycolysis, and for this reason, silenced PTEN is capable of activating the transcriptional factor SREBP [87], leading to an overexpression of fatty acid synthase, and in the same manner, their β-oxidation [88].

Regulation of steatosis by PTEN alters the tumor microenvironment as well. Deletion of PTEN leads to increased lipogenesis and lipid deposition. In hepatocytes, such lipid accumulation results in hepatocyte death, which sets up a niche activation signal. This then leads to tumor-initiating cell proliferation and tumorigenesis. However, the mechanisms by which PTEN regulates lipid and mitochondrial metabolism are not fully understood. Future studies to understand the molecular signals that PTEN controls to regulate these cellular functions are necessary for cancer treatment [89].

#### 4.3.3. Liver Kinase B1

The tumor suppressor liver kinase B1 (LKB1) is a serine/threonine kinase that interacts with the pseudokinase STE20-related kinase adaptor alpha (STRAD alpha) and with mouse protein 25 (MO25), and once activated, it phosphorylates and regulates AMPK, which has essential functions in cell metabolism, cell growth, and survival, and is frequently mutated in cancer [90]. During nutrient deprivation and hypoxia, high intracellular adenosine monophosphate (AMP)/ATP ratios activate the LKB1-AMPK pathway, since it acts as a sensor of energy availability. As a result of activation, they increase the catabolic processes that generate ATP (glycolysis and oxidation of fatty acids) and decrease the speed of the anabolic pathways that consume ATP (synthesis of proteins, cholesterol, and fatty acids) [91].

Although the influence of LKB1 on cell metabolism is not fully defined, studies show that its loss induces a metabolic change in proliferating cells dependent on HIF-1, which accumulates in cells deficient in LKB1, resulting in greater absorption and utilization of glucose and glutamine [92]. On the other hand, it has also been shown that functional expression of LKB1 allows for more effective oxidation of pyruvate, glutamine, and fatty acids than LKB1-deficient cells [93]. Similarly, another study demonstrated the importance of LKB1 in controlling excessive ROS levels during glucose deficit by inducing Nrf2, which induces antioxidant enzymes, such as heme oxygenase 1 (HO1) and NAD(P)H:quinone oxidoreductase1 (NQO1) [94].

#### 4.3.4. Tuberin

Tuberin, or tuberous sclerosis complex 2 (TSC2), is a negative regulator of mTOR signaling. Therefore, the phosphorylation of TSC2 by AKT, or other kinases, leads to its inactivation and, consequently, to activation of the mTOR complex [95]. Therefore, the loss or mutation of TSC2 in some types of cancer is associated with increased mTOR activity and decreased AKT phosphorylation [96]. The PI3K/AKT/mTOR pathway plays an important role during tumorigenesis and mTOR is a crucial regulator during this process, thus its increased activity is a central point of importance during metabolic reprogramming, proliferation, and survival [97].

During TSC2 mutation, there is an increase in HIF-1, which often results in a high glucose consumption rate [98]. Furthermore, mTOR controls fatty acid oxidation and provides substrates to generate energy through the TCA cycle and oxidative phosphorylation and meet the nutritional demands of cancer cells during glucose deficiency [99]. Another process regulated by mTOR is autophagy, a critical determinant of metabolic homeostasis and survival. During hyperactivation of mTOR, a product of TSC2 mutation, autophagy is decreased [100]. Its deregulation leads to PPP alterations, and consequently, the accumulation of mitochondrial ROS [101].

### 4.4. Oncogenes

The MYC oncogene belongs to a family called “supertranscription factor” that regulates the transcription of 15% of the genome [102]. In carcinogenesis, MYC is capable of inducing a glycolytic phenotype through an increase in the GLUT1 transporter and various enzymes, including HK2, PKF, enolase 1 (ENO1), PKM2, and LDHA. On the other hand, it increases glutamine catabolism to support the TCA cycle through increased expression of the glutamine transporter ASCT2, overexpression of the enzyme GLS, and a decrease in GS [103].

Increased glycolytic flux coupled with glutaminolysis ensures a supply of substrate for anabolic pathways. Regarding protein synthesis, in which MYC plays a central role for supporting cancer cell proliferation [104], key roles have been identified for proteins and enzymes involved in lipid metabolism linked to oncogenic levels of MYC, such as fatty acid transporter CD36, various lipases (LPL) and kinases that contribute to fatty acid oxidation; however, MYC is also closely related to SREBP1, which regulates lipogenesis [105].

The overexpression of MYC is directly related to telomerase reactivation and/or telomerase reverse-transcriptase (TERT) upregulation. TERT is required for the regulation of MYC ubiquitination and stabilization, as well as its binding to target promoter genes through stabilization of the oncogene in chromatin, resulting in an increase of MYC levels at high- and low-affinity promoters. MYC behaves as a nexus that unifies different signaling pathways and cellular modulators that control TERT expression and telomerase activity; in response to multiple cellular and environmental signals, these include NF-KB, Wnt/B catenin, and MAPK signaling pathways, members of the signal transducer and activator of transcription (STAT) family, specificity proteins (Sp) family, and p53. Hence the feed-forward activation loop between MYC and TERT is functionally critical in the process of oncogenesis [106,107].

On the other hand, Kirsten-ras (KRAS) oncogene mutations have been shown to promote metabolic reprogramming by modifying metabolism via anabolic pathways, starting with increased absorption of glucose, glutamine, and fatty acids. Elevated glycolysis is present, and the immediate increase of GLUT1, thanks to the activation of MAPK, regulates transcription directed by MYC [108]. In addition, KRAS increases the expression of glycolytic enzymes and the PPP enzyme ribose-5-phosphate isomerase (RPIA) to increase nucleotide synthesis and maintain tumor proliferation [109].

Moreover, KRAS also stimulates other processes to overcome nutritional demand, such as macropinocytosis and autophagy during conditions of nutrient deprivation. Both pathways provide a set of amino acids, lipids, nucleotides, and glucose that will be used to synthesise new macromolecules [110]. Studies have shown that during macropinocytosis, there is a great absorption of albumin, and during its decomposition, glutamine is released, an amino acid of great importance in cancer [111]. At the same time, KRAS stimulates another alternative pathway for glutamine uptake, which is through the regulation of glutamic-oxaloacetic transaminase (GOT) and GLUD1 [112].

Mutations of the EGFR activate survival, pro-invasive pathways, and play a significant role in regulating cell metabolism, inducing enhanced glycolysis, PPP, pyrimidine biosynthesis, and redox metabolism during tumorigenesis.Metabolic reprogramming by EGFR is carried out through the PI3K/AKT/mTOR pathway, which increases MYC and induces enzymes (e.g. HK2) and glucose transporters (e.g. GLUT1) [113]. On the other hand, EGFR increases the synthesis of monounsaturated fatty acids (MUFA) by phosphorylating SCD1 and SREBP-1 [114,115], and this may lead to SREBP-1-dependent survival through the regulation of LDLR [51].

Alterations of anaplastic lymphoma kinase (ALK), such as rearrangements, mutations, and genomic amplification, have been involved in malignancies. Studies indicate the presence of increased glucose metabolism with a potential metastatic phenotype in the echinoderm microtubule-like protein 4 (EML4)-ALK rearrangement [104]. One of the reasons could be the overexpression of HK2, which mediates the metabolic effects of EML4-ALK, through the induction of HIF-1 by transcriptional and post-transcriptional mechanisms [116].

In spite of the fact that activation and overexpression of oncogenes can stimulate cell proliferation, they can also cause genetic stress that results in senescence, a phenomenon known as oncogene-induced senescence (OIS). This type of senescence has anti- as well as protumorigenic effects, depending on the conditions. Thus, the senescence-associated secretory phenotype (SASP) can contribute or not to the progression of tumors depending on the factors secreted. During cancer development, secreted factors such as VEGF, IL-8, IL-6, and the chemokine (C-X-C motif) ligand 1 (CXCL1) promote tumor progression by stimulating angiogenesis. In turn, CXCL1 and ECM degrading enzymes participate in the recruitment of immune cells to form a microenvironment around senescent cells and escape immune surveillance programs [117,118].

## 5. A Brief Analysis of Therapies Modulating Cell Metabolism

Due to the importance of metabolic reprogramming in tumor replication and survival, it represents a striking therapeutic target that should be taken into account when developing possible cancer treatments. Over the years, different drugs have been studied that alter the cellular metabolism of cancer cells and could help fight the disease [82,119,120]. The following section summarizes key clinical evidence for metabolic reprogramming as a therapeutic target for cancer (Table 1).

### 5.1. mTOR Inhibitors

The mechanistic target of rapamycin (mTOR) is a serine/threonine kinase that is part of two protein complexes: mTORC1 and mTORC2. mTORC1 is regulated by growth factors, nutrients, as well as stress, and it promotes protein, lipid, nucleotide synthesis, glycolysis, and the PPP through the expression of genes involved in each, as well as inhibiting autophagy, among others [127]. In contrast, mTORC2 is upregulated by PI3K/Akt and inhibited by mTORC1, which promotes cell proliferation and survival through enzymatic phosphorylation, where AKT is its main substrate [128].

In different types of cancer, there may be aberrant activation of both mTORC1 and mTORC2 due to mutations in the genes that encode these proteins [127], or in closely related pathways, such as PI3K or Akt; thus, it represents one of the most frequently hyperactive proteins in cancer, playing an important role in its pathogenesis [129]. Hence, drugs targeting mTOR represent a novel theraupetic approach and hold promise in cancer therapy.

#### Rapalogs

Rapamycin is an immunosuppressive drug that regulates cell proliferation and growth by inhibiting mTOR—specifically mTORC1—to alter the metabolism of cancer cells [130]. However, the solubility and pharmacokinetics of rapamycin leave much to be desired, which is why analogous drugs of rapamycin, or rapalogs, have been developed with improved pharmacological properties, including everolimus, temsirolimus, and ridaforolimus [131].

Rapalogs can be effective in treating different types of cancer, which is why they have been approved for the treatment of some of them [132]. For example, temsirolimus has been approved by the US Food and Drug Administration (FDA) for the treatment of advanced renal cell carcinoma [133], and in the European Union, for the treatment of relapsed/refractory mantle cell lymphoma (MCL) [134]. Everolimus was approved by the FDA for the treatment of advanced hormone receptor positive and HER2 negative breast cancer in addition to the treatment of some non-functional neuroendocrine tumors, among others [135].

However, the use of these drugs is by no means a perfect alternative, and side effects are often observed in monotherapy [136], which have even been surpassed by those of better supportive treatment in advanced gastric cancer [137]. This may be due to the fact that although these drugs reduce the growth and aggressiveness of tumor cells through the PI3K/AKT/mTOR signaling pathway [138], rapalog monotherapy may lead to drug resistance. Since rapalogs are only cytotoxic at very high levels, they function as cytostatic agents at safe concentrations [139]. Therefore, the combined use of rapalogs with conventional cytotoxic drugs is currently under study [140].

### 5.2. ATP-Competitive mTOR Inhibitors

Among the ATP-competitive mTOR inhibitors are sapanisertib (TAK-228, MLN0128 or INK128) [141,142,143], AZD8055 [144], AZD2014 [145], among others. Unlike rapalogs, this group of drugs inhibits both mTORC1 and mTORC2, acting directly on their catalytic sites [146], thus inhibiting protein synthesis more drastically, arresting the cell cycle [147], and producing selective apoptosis in some cancer cells [148].

Regarding clinical evidence, phase I studies have shown that TAK-228 and AZD8055 have an acceptable safety profile with promising antitumor properties for some solid tumors [148,149]. Moreover, in a phase II clinical study conducted by Basu et al. [121] in patients with different types of cancer, AZD2014 showed good tolerability and clinical efficacy as a single agent.

However, in a phase II study by Graham et al. [122] that evaluated the effect of MLN0128 in patients with metastatic prostate cancer, the effects of MLN0128 were found to be limited, possibly because the dose was reduced in some patients due to drug toxicity. Similarly, in a phase II study in patients with relapsed or refractory diffuse large B-cell lymphoma (DLBCL), treatment with AZD2014 did not show a better result than treatment with rapalogs [150].

Thus, despite the promising results of both in vitro and in vivo preclinical studies [151,152], contradictory results in different clinical studies indicate that these drugs should be investigated further to better understand their possible role in cancer treatment.

#### Dual PI3K/mTOR Inhibitors

This group of drugs acts both on the catalytic site of mTOR and the p110α subunit of PI3K, producing better effects than rapalogs by avoiding negative feedback via the mTOR-ribosomal protein S6 kinase beta-1 (S6K1)-insulin receptor substrate 1 (IRS-1) loop that increases the activity of the PI3K pathway [153]. Some of the drugs belonging to this group are PI-103, NVP-BEZ235, WJD008, PF-04691502, PF-502, and PKI-402.

Preclinical evidence for these drugs has been promising, as reflected in the study by Bresin et al. in which PF-502 showed potent anticancer activity against cutaneous T-cell lymphoma, both ex vivo in patient-derived cells, especially those that had reduced expression of PTEN, and in vivo in animal models [154]. Similarly, in another study by Hu et al., PKI-402 inhibited proliferation, altered mitochondrial functions, and induced apoptosis in human ovarian cancer epithelial cells with PIK3CA gene mutations [155]. Likewise, the study carried out by Wu et al. showed BEZ235 suppressed tumor growth in lung cancer, both in vitro and in vivo, and enhanced the effects of cisplatin chemotherapy [156].

For its part, the clinical evidence is still limited, and the studies published to date have not been very encouraging. Although some of these drugs have shown manageable safety profiles with modest antitumor activity for some types of cancer [157,158,159], other studies have shown no benefit compared to rapalogs, as evidenced by a phase II clinical study conducted by Salazar et al. in which BEZ235 was compared to everolimus in patients with advanced pancreatic neuroendocrine tumors. The results showed that treatment with BEZ235 was not more effective than everolimus and, in addition, was less tolerated [160]. Likewise, it has been observed that a common side effect of treatment with dual PI3K/mTOR inhibitors is hyperglycemia, although it can usually be treated conventionally and does not require treatment discontinuation [161].

### 5.3. Glutaminase Inhibition

GLS is important for maintaining antioxidant defense in a cancer cell since it mediates the transformation of glutamine into glutamate, so its inhibition can produce a decrease in GSH and an increase in oxidative stress, which may result in the death of cancer cells [162]. Thus, it is a promising therapeutic strategy [163], which is why various drugs capable of inhibiting GLS have been developed, including 6-Diazo-5-oxo-L-norleucine (DON), Bis-2-Sulfide (5-phenylacetamido-1,3,4-thiadiazol-2-yl) ethyl (BPTES), CB-839, 968, JHU-083, fisapubescin, etc [164].

Regarding preclinical evidence, it has been possible to discern how the inhibition of GLS can reduce tumor growth, as shown in the study by Emadi et al. [165], which found that BPTES, the first identified allosteric inhibitor of GLS1, slowed the growth of IDH-mutated primary acute myeloid leukemia cells. However, the low solubility of BPTES reduces its pharmacological potential; therefore, it has been modified to improve its pharmacological properties while maintaining its GLS inhibitory capacity, resulting in promising molecules, such as CB-839 [166]. Ren et al. demonstrated that CB-839 could inhibit the growth of osteosarcoma cell lines and and an orthotopic xenograft in vivo, and when combined with metformin, its effect was enhanced, significantly inhibiting the growth of a primary tumor in vivo and reducing its metastatic progression [167].

Clinical evidence is still scarce, and further investigation is needed, since various phase I and II clinical trials of DON in patients with different types of cancer have demonstrated considerable toxicity, mainly gastrointestinal, and a null tumor response [168]. A phase II clinical study conducted by Mueller et al. showed that combined treatment with DON and the glutamine-reducing enzyme PEG-PGA was well tolerated and showed activity in advanced colorectal and lung cancer, leaving the door open for further research on this drug [123]. Similarly, other molecules have been shown to have less toxicity, such as CB-839, as evidenced by a phase I clinical study where CB-839 was administered in combination with cabozantinib to patients with metastatic renal cell cancer with encouraging clinical activity and good tolerability [124]. Although limited information is currently available on the clinical effectiveness of this group of drugs, there are several ongoing clinical trials that could shed light on the matter [169,170].

### 5.4. Biguanides

Biguanides are a group of drugs used as antidiabetics, among which metformin stands out as the most used [171], that have also been studied as a possible treatment for other pathologies, including cancer [172]. The potential effects of these drugs on cancer have generated interest due to preclinical studies, where in vitro and animal models have shown that metformin can reduce tumor proliferation and survival of different types of cancer, such as ovarian, breast, and colon cancer [173]. Although the pathways have yet tobe fully elucidated, they may involve mTORC1 suppression, AMPK activation, reduction in ROS production, modulation of A1 adenosine receptor expression, and reduction in serum insulin levels, IGF-1, and IGF-2, among others [174].

Regarding clinical evidence, in 2005 Evans et al. used record linkage databases developed in Tayside, Scotland, a diabetes clinical information system, and a database of dispensed prescriptionsto demonstrate that metformin may reduce the risk of cancer in patients with type 2 diabetes [175]. Subsequently, various studies and meta-analyses have observed a reduction in the incidence of cancer, especially colon and pancreatic cancer, in patients with type 2 diabetes treated with metformin compared to those treated with other antidiabetic drugs [176,177]. However, it has not been possible to verify whether the beneficial effect of metformin in these types of cancer in diabetic patients is due to the positive effects of metformin or the negative effects of other therapies. Furthermore, the characteristics of patients treated with metformin may be different from those who were given another drug [178].

### 5.5. HER2 Inhibitor

An oncogenic receptor tyrosine kinase known as HER2 is strongly expressed in certain cancers. HER-2 has been shown to prevent cancer cells from producing cytokines by potently inhibiting cGAS-STING signaling [179]. Therefore, new strategies against HER2 are under study, as is the case for KU004 [180]. This drug has been associated with the inhibition of a metabolic pathway known as PEI3K/Akt after inhibiting the Warburg effect by blocking HER2, making it an attractive therapeutic agent for the clinical treatment of HER2+ cancer [181]. It should be noted that one negative side effect could be drug resistance; however, new-targeted therapies to attenuate this resistance are still under study [182,183,184,185].

### 5.6. HIF-1 Inhibitor

In addition to its fundamental role in metabolic reprogramming, HIF-1 is involved in chemoresistance, metastasis, angiogenesis, and immunosuppression in cancer cells; therefore, it is emerging as an important therapeutic target [186]. Some drugs have been developed that can intervene in the action of HIF-1, such as SC-144, a pyrroloquinoxaline derivative with broad-spectrum anticancer activity [187], which can reduce HIF-1α expression and increase levels of hypoxia-inducible antisense factor (HIF-1α-AS) after prolonged exposure, and may explain, at least in part, its antitumor activity [188]. In a similar manner, EZN-2698, an antisense oligonucleotide, is another drug that inhibits HIF1α mRNA expression, producing a reduction in tumor progression both in vitro and in vivo [189].

In addition to this, there are natural compounds that show promise for the treatment of cancer, such as crocin, one of the bioactive compounds in saffron, which can reduce the expression of HIF-1α in gastric cancer tumor cells, thus reducing the migration, invasion and EMT of gastric cancer cells [125,190]. Another natural compound, ginsenoside 20(S)-Rg3 extracted from Panax ginseng, promotes the degradation of HIF-1α in ovarian cancer by targeting miR-519a-5p resulting in the inhibition of EMT and suppression of HK2 expression, a crucial enzyme in the Warburg effect [126,191].

### 5.7. Ion Channels as Drug Targets

Ion channel inhibition is a pharmacological strategy studied for its potential key feature of arresting tumor growth and metastasis [192]. In this context, several ion channel modulators are already in clinical use or are currently being tested in clinical trials, such as Cltx, which inhibited glioma cell migration in vitro and in vivo in mice [193,194,195].

Another potential therapeutic target is the regulation of ion channels by Ca^2+^, since multiple channels commonly implicated in glioma biology require high levels of intracellular Ca^2+^ for activation. In the same vein, the feasibility of targeting tumor cell ion channels with compounds such as synthetic chlorotoxin (TM-601) [196,197] has been studied since it represents a potentially important targeting agent that causes many cancers to internalize ClC-3, which inhibits glioma cell migration [198].

On the other hand, persistent current blockers of voltage-dependent Na channels, such as ranolazine (“Ranexa”) and riluzole (“Rilutex”), have been classified within the group of antimetastatic agents [199,200]. Although these drugs are already used clinically for other pathologies and suggest a good pharmacodynamic profile, channel inhibitors are limited by side effects, such as prolonged QT syndrome, severe cardiac arrhythmia, and sudden cardiac death [201].

The challenge in using this type of therapy includes identifying the differences between tumor types and the involvement of multiple ions in each particular case. In addition, the adverse effects are striking, since channel blockers will affect healthy and mutated cells alike [202].

### 5.8. Others Therapies

Other therapeutic strategies include compounds known to block glucose metabolism, such as 2-deoxyglucose (2-DG), or glucose transporters [203,204], such as phosphonoacetohydroxamate (PHAH), and SF-2312, which can additionally inhibit enolase activity (ENO). Isoform 1 of monocarboxylate transporters (MCT) can be inhibited by the compound AZD3965. Targeting altered glucose metabolism to regulate metabolic reprogramming is another therapeutic alternative that has been studied; however, high glucose uptake is not a unique feature of tumors and offers a hypothetical explanation for the relative lack of success in treating cancer by directly targeting glucose metabolism [43].

Other drugs such as methotrexate, pemetrexed, 6-mercaptopurine, capecitabine, gemcitabine, and leflunomide are considered great therapeutic agents for targeting key enzymatic points in metabolic reprogramming; however, this group of drugs has been studied more extensively by other authors [43].

## 6. Future Perspectives

Conventional therapies such as chemotherapy, radiotherapy, and surgery are standard in cancer treatment; however, the multiple responses to them and advances in cancer cell behavior demonstrate the importance of exploring alternative treatment options. The use of cancer metabolism as a therapeutic target is increasingly promising and suggests the way forward for the development of effective therapies for cancer eradication. Nevertheless, the creation of drugs that inhibit metabolism will not guarantee their success; thus, it will be equally important to refine the processes of identifying patients who will best respond to these types of therapies. Although most of the metabolic targets of the previously described therapies are overexpressed in cancer, the dependence on these metabolic pathways varies from one person to another. Hence, a better understanding of metabolic dependencies in specific tumor tissues is key to defining the aspects of metabolism that most limit tumor growth; only then can vulnerabilities be explored to provide better cancer treatments in the near future.

## 7. Conclusions

The description of the highly complex interactions between the main deregulated metabolic pathways, the increase or decrease of crucial enzymes, the mitochondrial metabolism, and REDOX regulation have been extensively studied. The results have rendered new approaches for novel anticancer therapeutic strategies that target the essential pathways of cancer cells. Some of those surveyed have been: metformin, mTOR inhibitors, glutaminase inhibitors, and ion channels as drug targets. However, developing new technologies and improving experimental methods is still necessary to further explore the entire landscape of metabolic alterations and their effects that will leading to more accurate anticancer strategies based on this emerging hallmark.

## Figures and Tables

**Table 1:** Summary of key clinical evidence for metabolic reprogramming as a therapeutic target for cancer.

Authors (REF)	Treatment	Inhibitor Target	Methodology	Results
DeCensi et al. [119]	Metformin	Insulin-lowering agent.	Meta-analysis combining 11 observational studies and trials (N = 4042) focused on evaluating the effects of metformin on cancer.	A 31% reduction in cancer incidence or mortality was observed in subjects taking metformin compared with other antidiabetics (SRR, 0.69; 95% CI, 0.61–0.79).
Andronesi et al. [120]	IDH305	Mutant-selective allosteric high affinity IDH1 inhibitor.	Phase I clinical trial of IDH305.	A rapid decrease of 2HG levels by 70% (CI 13%, *p* = 0.019) after 1 week of treatment. Importantly, inhibition of mutant IDH1 may lead to reprogramming of tumor metabolism, suggested by simultaneous changes in glutathione, glutamine, glutamate, and lactate.
Basu et al. [121]	AZD2014	Dual m-TORC 1/2 Inhibitor.	Phase II clinical trial where 56 adults were treated with AZD2014 orally twice daily with doses of 25 to 100 mg.	Two PRs were evidenced in the study. The first was a patient with acinar pancreatic cancer with KRAS, PDGFRA, APC, ERB4, KIT, and FBXW7 mutations. The second to respond was a breast cancer patient with HRAS, NRAS, TP53 and ERBB2 mutations.
Graham et al. [122]	MLN0128	Dual m-TORC 1/2 Inhibitor.	Phase II clinical trial focused on evaluating the efficacy and safety of MLN0128 in mCRPC at a dose of 4 mg for a median time of 11 weeks (range = 3–30) in 9 subjects.	The best response was stable disease. All subjects showed an increase in PSA during treatment (159%) and no decreases in cancer cell counts after treatment. Correlative studies of biopsy samples before and after treatment indicate limited inhibition of AKT phosphorylation, 4EBP1, and eIF4E activity.
Mueller et al. [123]	6-diazo-5-oxo-L-norleucine (DON)	Glutamine antagonist and inhibits irreversibly the purine/pyrimidine synthesis.	Phase IIa multicenter study evaluating the safety and clinical activity of the glutamine-reducing enzyme PEG-PGA in combination with DON in 55 patients treated with 120 IU/m^2^ of PEG-PGA once a week, and 140 mg/m^2^ DON twice a week between 1 and 379 days.	After the second infusion, glutamine levels were reduced by 10% and remained reduced in >90% of patients. Among the patients with metastatic colorectal cancer, 1 PR was found, 9 free of progression at 3 months, and 5 free of progression at 5 months.
Meric-Bernstam et al. [124]	CB-839	Glutaminase Inhibitor.	Escalating doses of CB-839 (600–800 mg PO BID) plus Cabo (60 mg PO QD) were evaluated using a 3+3 design. Tumor response was assessed per RECIST 1.1 every 8 weeks.	CB-839 plus Cabo showed encouraging clinical activity and tolerability in heavily pretreated mRCC patients, with response rates (50% ORR, 100% DCR) comparing favorably to historical Cabo monotherapy.
Zhou et al. [125]	Crocin	Inhibits the migration, invasion, and epithelial-mesenchymal transition of gastric cancer cells via miR-320/KLF5/HIF-1α signaling.	Preclinical study	Crocin inhibits the EMT, migration, and invasion of gastric cancer cells, and this activity is mediated by miR-320/KLF5/HIF-1α signaling
Liu et al. [126]	Ginsenoside 20(S)-Rg3	Reduces the expression of HIF-1α by activating the ubiquitin-proteasome pathway to promote HIF-1α degradation.	In vivo and in vitro preclinical study	Ginsenoside 20(S)-Rg3 potently blocks hypoxia-induced EMT of ovarian cancer cells

CI, confidence interval; PR, partial response; mCRPC, metastatic castration resistant prostate cancer; PSA, prostate specific antigen; IDH1, isocitrate dehydrogenase 1; HIF-1α, hypoxia-inducible factor 1α.

## Data Availability

Not applicable.
